# Medical ward round competence in internal medicine – an interview study towards an interprofessional development of an Entrustable Professional Activity (EPA)

**DOI:** 10.1186/s12909-016-0697-y

**Published:** 2016-07-11

**Authors:** Teresa Wölfel, Esther Beltermann, Christian Lottspeich, Elisa Vietz, Martin R. Fischer, Ralf Schmidmaier

**Affiliations:** 1 Institut für Didaktik und Ausbildungsforschung in der Medizin, Klinikum der LMU München, Ziemssenstrasse 1, 80336 Munich, Germany; 2 Medizinische Klinik und Poliklinik IV, Klinikum der Universität München (LMU), Ziemssenstrasse 1, 80336 Munchen, Germany

**Keywords:** Competency, Curriculum development, Entrustable professional activity, Internal medicine ward round, Teaching methods

## Abstract

**Background:**

The medical ward round is a central but complex activity that is of relevance from the first day of work. However, difficulties for young doctors have been reported. Instruction of ward round competence in medical curricula is hampered by the lack of a standardized description of the procedure. This paper aims to identify and describe physicians’ tasks and relevant competences for conducting a medical ward round on the first day of professional work.

**Methods:**

A review of recent literature revealed known important aspects of medical ward rounds. These were used for the development of a semi-structured interview schedule. Medical ward round experts working at different hospitals were interviewed. The sample consisted of 14 ward physicians (*M* = 8.82 years of work experience) and 12 nurses (*M* = 14.55 years of work experience) working in different specializations of internal medicine. All interviews were audiotaped, fully transcribed, and analyzed using an inductive-deductive coding scheme.

**Results:**

Nine fields of competences with 18 related sub-competences and 62 observable tasks were identified as relevant for conducting a medical ward round. Over 70 % of the experts named communication, collaborative clinical reasoning and organization as essential competences. Deeper analysis further unveiled the importance of self-management, management of difficult situations, error management and teamwork.

**Conclusion:**

The study is the first to picture ward round competences and related tasks in detail and to define an EPA “Conducting an internal medicine ward round” based on systematic interprofessional expert interviews. It thus provides a basis for integration of ward round competences in the medical curricula in an evidence based manner and gives a framework for the development of instructional intervention studies and comparative studies in other medical fields.

**Electronic supplementary material:**

The online version of this article (doi:10.1186/s12909-016-0697-y) contains supplementary material, which is available to authorized users.

## Background

### Aim of this study

Conducting an internal medicine ward round is a central, but complex, daily activity in internal medicine inpatient care [1]. Ward rounds often are the only time in a day when physicians, nurses and patients get together to jointly exchange information regarding diagnosis and treatment. Thus, ward rounds highly contribute to the quality of patient care [2, 3]. Ward rounds are characterized by collaborative clinical reasoning of physicians and nurses with regard to evidence-based patient care, physicians’ and nurses’ perspectives as well as patients’ social and emotional situation [2]. Apart from this, ward rounds constitute a valuable component in both undergraduate and graduate medical education that aims at imparting clinically relevant knowledge [4].

Directly after their graduation, physicians are responsible for carrying out this complex task. Research reports that physicians struggle in overtaking this task and transitioning to the workplace’s demands successfully [5–7].

With the aim of facing those problems important research was conducted during the last years. It could be shown, that conducting good ward rounds is a complex teachable competence and thus should be integrated in the medical curricula. Simulation-based ward round trainings were identified to be a realistic opportunity to ease the transition from ward round participant to ward round leader, e.g. by gaining important feedback to improve potential deficits [8]. Krautter et al. [7] showed positive effects of structured on-ward-round supervision on students’ satisfaction as well as on their performance concerning patient education, scheduling of diagnosis and documentation. Further it became clear that there is a need for a stronger focus on interprofessional teamwork in health care education, especially for nurses and physicians, to meet the standard of both professions [9–11]. Kiesewetter and Fischer [12] provided an approach for the needed teamwork training with the “Teamwork Assessment Scale”, an instrument that allows the evaluation of behavioural components and can be applied during simulated ward round trainings.

Knowing that a complex activity like conducting a ward round could be taught, the need for a guiding and assessment tool became obvious. Some important approaches are already made such as “The Basel Standard for Doctorʼs Visits” [3] or the validated checklist from Norgaard et al. [13]. However, there are no published competence-based educational objectives, developed in a structured manner with an interprofessional team, to face the standards of both, nurses’ and physicians’ profession, and can further be integrated in the competence-based medical education. Although partial aspects of ward round competences are already integrated in the curriculum (e.g. patient-communication skills), a specific, competence-based definition of educational objectives is still missing. This hampers further development of teaching and assessment methods in this research field and that is why we aim to develop a competence-based assessment guideline for ward rounds using a structured, interprofessional interview study for data collection.

### Entrustable Professional Activities (EPA) – a connection between theory and practice

Ten Cate et al. [14, 15] defined the readiness for unsupervised practice as a significant milestone and a central goal of medical education.

As the medical workplace is characterized by a high complexity, the CanMEDs, by the Royal College of Physicians and Surgeons of Canada, provided a helpful basis for characterizing competences around the following six roles of the physician: communicator, collaborator, health advocate, scholar, professional, manager, medical expert [16].

Ten Cate [17] translated such a theoretical competence framework in the concept of “Entrustable Professional Activities” (EPAs). EPAs directly refer to professional practice and comprehend tasks and responsibilities a physician should take over when acting in a particular professional situation. Once a learner has achieved sufficient competence in a field through participating in a form of medical education, these tasks can be entrusted to be performed in an unsupervised way [18]. Besides the possibility to implement competences in a daily working and teaching routine, EPAs could simplify curriculum design and implementation of medical training [17, 19–21]. The decision when an activity can be entrusted to the trainee depends on many different factors. To facilitate this decision, it is recommended to use levels of supervision (LoS) for the assessments [15]. LoS describe how much supervision the specific trainee needs for fulfilling the activity. This can be the need for the supervisor to be present and observe the trainee during the whole activity (LoS 1) or the fact, that the trainee himself is ready to provide supervision to others concerning this specific activity (LoS 5). With linking an EPA to an entrustability scale like the LoS the personal clinical judgment can directly be transferred to a general scale without the need for a translation in codes. This can increase the reliability of the assessments and the amount of well-constructed feedback [22].

Therefore we referred to the EPA approach to face the complexity of internal medicine ward rounds and the development of a solid assessment.

Prior studies already addressed the development of EPAs in different fields of medical education but mainly referred to standardized tasks with a consistent structure and clear responsibilities (e.g. patient handover [14]). The ward round, however, represents a complex activity that is assumed to require more competences and consequently more tasks to master the situation successfully. While former approaches used small group discussions to define competences and activities relevant for mastering a situation successfully, a more sophisticated procedure was necessary for this study which accounts for (i) the various requirements imposed on physicians, and (ii) an interprofessional perspective.

A three step approach was used to develop a best practice model in an EPA manner:

1. Which competences did prior research suggest as relevant for conducting ward rounds?
  The qualitative analysis of the interview transcripts should
2. Which competences and sub-competences do ward round experts regard as relevant for a young professional conducting an internal medicine ward round?
  The results should provide the framework for the interview structure to answer the second research question.
3. How can the competences and sub-competences be integrated into an EPA “Conducting an internal medicine ward round”?
  provide the scientific framework for the third research question.

## Methods

### Review of the literature for development of the interview schedule

In advance to the main study a review of recent literature was conducted aiming to identify domains of competences relevant for an internal medicine ward round as suggested by prior research. Therefore following key words were included: “ward round”/“round”/“bedside round” in connection with “competence”/“competency”/“skill”/“ability”. The electronic databases PsychInfo, PsychIndex, PubMed and Web of Science were used for the research. In a first step 1760 articles were found that were published after 01.01.1990 and were peer reviewed. In a second step doublings were removed and titles of the papers were screened concerning relevance. The abstracts of the 57 remaining articles were screened. Articles were included when (1) focusing on internal medicine ward rounds and (2) pointing out relevant competences and (3) when based on empirical data or on literature review. Only German and English articles were included. Articles were excluded when the teaching environment of ward rounds was the only focus of the article. In the end ten papers were found to be relevant for the development of the interview schedule. Excerption of the ten articles was summarized and used for the development of the interview schedule.

### Interviews with experts to identify internal ward round competences

An interview study was conducted using a semi-structured interview schedule for data collection.

#### Development of the interview schedule

To generate a holistic picture of ward round related tasks and competences the interview schedule was subdivided into three parts:

(i) Questions concerning process and structure of the ward round, to generate a better understanding of the general framework the ward round physician has to be aware of and to generate a common understanding of the ward round the interviewee was referring to.
  Exemplary questions:
  *How long does the ward round typically take? (in total/per patient)*
  *How does the ward round usually end? (*e.g. *concluding discussion, immediate disbandment)*
(ii) General questions about ward round related tasks and competences, to identify relevant domains of competences from the experts’ point of view.
  Exemplary questions:
  *What function does the ward physician have in the course of the ward round? Which specific tasks belong to that function?*
(iii) Specific questions concerning ward round related domains of competences as stressed in prior research, to enhance the understanding of their relevance.
  Exemplary questions:
  *How would you describe the physician-patient communication in the course of the ward round? How and to what extent you adapt your language use to the patient and/or the ward round team? (If interviewee can’t answer the question: Could you give an example in which adaption of the language is necessary?)*

The focus of the questions in all three parts was on competences relevant for young professionals. With the usage of open questions in all parts, we allowed the interviewees to add further aspects [23].

Questions in interview part (i) and (iii) focused on the identification of the practical application as well as sub-competences and observable tasks of competences identified in prior research. In part (i), organizational competences are interrogated separately since they were seen as an easy introduction to the interview.

The aim of the unstructured questions regarding ward round competences and belonging tasks in interview part (ii) was to identify further domains of competences and to minimalize a potential bias by the interview questions.

#### Setting and sample

The interview study was performed with a total of 26 physicians and nurses. They were selected from different care levels (university hospitals (11), academic teaching hospitals (15)) and different specializations of internal medicine (General Internal Medicine (7), Cardiology (2), Gastroenterology (3), Haematology/Oncology (7), Immunology/Rheumatology (2), Nephrology (2), Endocrinology (2), Alternating (1)) representing the broad field of internal medicine and the different perspectives of ward round participants. Thus, the interprofessional aspect of ward rounds was reflected and both perspectives were considered for the EPA development. Further the broad fields of internal medicine ward rounds and the various challenges young physicians are facing when conducting ward rounds were covered.

Participants were chosen based on (i) the amount of their clinical experience with a minimum of four years to possess a realistic view of typical and unusual rounds and (ii) their function as ward physicians or a comparable assigned responsibility for nurses (e.g. nurse manager, practical instructor in nursing). The sample comprised 14 physicians having *M* = 8.82 years of work experience (*SD* = 7.50) and 12 nurses having *M* = 14.55 years of work experience (*SD* = 4.80). Physicians reported to participate in *M* = 4.36 (*SD* = 1.71) and nurses in *M* = 5.1 (*SD* = 0.51) ward rounds per week, respectively.

#### Procedure

After piloting and adjusting the interview schedule accordingly, interviews were performed using the semi-structured interview schedule (Additional file 1: Data S1). Interviews lasted 42.52 min on average (SD = 12.28). All interviews were performed by the same scientist (first author) to ensure a standardized procedure. Interviews were audiotaped for data analysis. In accordance with the Declaration of Helsinki, the study was approved by the local ethics committee. Participation in the study was voluntary and based on informed consent.

#### Data analysis

Eighteen hours and thirty one minutes of audio data were transcribed and content analysis was performed [24] using MAXQDA 11. To ensure conformance and reliability, an inductive-deductive coding scheme was used. Eight (>25 %) randomly selected interviews were coded by two coders (first and second author) independently with 76 % agreement. The remaining interviews were coded by one coder (first author).

To identify ward round domains of competences, each interview was divided into two main parts. The first part included open questions about ward round tasks and related competences (interview schedule part ii) to identify general domains of competences from the experts’ point of view avoiding a potential influence of the interview questions.

The second part referred to more structured questions regarding the ward round framework and to specific ward round competences (interview schedule parts i and iii).

### Integration of the identified competences in an EPA model

For the development of a typical EPA structure [21] the gained information was restructured and overlapping elements were summarized. To ensure representativeness of the information incorporated in the EPA, categories were included when at least 25 % (*N* > 7) of participants, from both professions, mentioned it.

## Results

### Domains of internal medicine ward round competences as indicated by prior research

The first research question put an emphasis on the identification of domains of competences indicated in prior research. We identified 12 domains of competences in the literature that were frequently linked to internal medicine ward rounds (Table 1). These competences built the basis for the development of questions for the third section of the interview schedule. Accounting for difficulties in conducting ward rounds as reported in prior studies (e.g. [5]), questions that target difficult situations and possible reaction to these issues, as well as possibilities of how ward round competences can be acquired, were included.

**Table 1:** Review of the literature concerning domains of ward round competences

Domains of competences	References
Clinical skills	Norgaard et al. [13]; Tariq et al. [31]
Collaborative clinical reasoning	Amin et al. [32]; Herring et al. [25]; Kirkpatrick et al. [33]; Norgaard et al. [13]; Roy et al. [27]; Tariq et al. [31], Weber et al. [2]
Communication: physician-patient	Amin et al. [32]; Herring et al. [25]; Kirkpatrick et al. [33]; Norgaard et al. [13]; Roy et al. [27]; Tariq et al. [31], Weber et al. [2]
Communication: physician-team	Amin et al. [32]; Herring et al.[25]; Kirkpatrick et al. [33]; Norgaard et al. 2004 [13]; Roy et al. [27]; Tariq et al. [31], Weber et al. [2]
Empathy	Mercer & Reynolds [34]; Roy et al. [27]
Error management	Herring et al. [25]; O’Leary et al. [35]
Organization	Amin et al. [32]; Herring et al. [25]; Norgaard et al. [13]; Roy et al. [27]; Tariq et al. [31]; Weber et al. [2]
Patient-management	Amin et al. [32]; Norgaard et al. [13]; Tariq et al. [31]; Weber et al. [2]
Professionalism	Amin et al. [32]; Kirkpatrick et al. [33]; Roy et al. [27]; Tariq et al. [31]
Self-management	Amin et al. [32]
Teaching abilities	Claridge [4]; Herring et al. [25]; Kirkpatrick et al. [33]; Norgaard et al. [13]; Roy et al. [27]; Tariq et al. [31]
Teamwork	Amin et al. [32]; Herring et al. [25]; Norgaard et al. [13]; O’Leary et al. [35]; Roy et al. [27]; Tariq et al. [31], Weber et al. [2]

### Domains of internal medicine ward round competences as identified in interviews with experts

#### Domains of ward round competences as identified in the open interview part

By open questions 15 domains of competences relevant for conducting ward rounds were identified (Table 2). Both physicians and nurses stressed the importance of communication, collaborative clinical reasoning and organization, and claimed them as key competences. Only 29 % of the physicians and 25 % of the nurses mentioned teamwork as a relevant competence in this section of the interview. In addition to the competences that were already known from prior research 12 % of the experts named “leading the patient” and 8 % “social competences” as relevant ward round competences.

**Table 2:** Relative frequencies of internal medicine ward round competences named in free association or as answers to specific questions

Domain of competence	Total		Physicians		Nurses	
	(*n* = 26)		(*n* = 14)		(*n* = 12)	
	% O	% S	% O	% S	% O	% S
^a^Communication: physician-team/patient	96	100	93	100	100	100
^a^Collaborative clinical reasoning	77	100	86	100	57	100
^a^Organization	69	100	64	100	75	100
^a^Self-management	46	100	64	100	25	100
^a^Teamwork	27	100	29	100	25	100
^a^Management of difficult situations	19	100	29	100	8	100
^a^Error management	4	100	7	100	0	100
^a^Professionalism	27	92	29	86	25	100
^a^Empathy	54	85	64	71	42	100
^a^Patient-management	42	73	57	79	25	67
^a^Clinical skills	54	69	64	93	42	42
^a^Teaching and learning abilities	19	62	29	93	8	25
Medical knowledge	58	42	64	57	50	25
Communication: physician-relatives	0	15	0	14	0	17
Leading the patient	12	0	21	0	0	0
Social competences	8	0	7	0	8	0

*O* Open Percentage of experts who named the competence as answer to the open question concerning which competences/tasks/abilities are needed

*S* Structured: Percentage of experts who emphasised the competence as relevant in questions directly asking about the specific competence

^a^known from prior research

#### Domains of competences as identified in the structured interview part

Analysis of the more specific, literature-based questions revealed a high overlap of relevant competences with those named by the experts upon open questions. Again, communication, collaborative clinical reasoning and organization were frequently named. Additionally, the importance of self-management, teamwork, and error management was stressed by all experts (Table 2).

In addition to these aforementioned competences, interviewees added medical knowledge (42 %) and communication with patients’ relatives (15 %) as relevant ward round competences.

Analysis further revealed a general ward round structure that consists of preparation (62 %), consultation of patients (100 %) and debriefing (38 %). 93 % of the physicians and 92 % of the nurses stressed the importance of regular participation of nurses. However 79 % of the physicians and 75 % of the nurses claimed that this is not possible in their daily routine.

### Development of the EPA “Conducting an internal medicine ward round”

Building on the competences that were identified in the interview study, we realized the EPA “Conducting an internal medicine ward round”. Each competence was subdivided into several sub-competences and observable tasks, mentioned by the interviewees, were added. In case of substantial overlaps in the domains of competences, these were regrouped after discussion in the research group and consultation of literature. The resulting EPA comprehends nine essential competences which were sub-divided into 18 sub-competences and 62 observable tasks (Additional file 2: Data S2). For reasons of clarity and comprehensibility only a short and restructured version with competences and sub-competences is shown in Table 3.

**Table 3:** Description of the EPA “Conducting an internal medicine ward round” (short version) with competences, sub-competences and related CanMEDS roles

Discipline	Internal Medicine	
Title	Conducting a ward round in internal medicine	
Description	Conducting the daily ward round in an internal medicine department, starting with the preparation.	
CanMEDS domains of competence	Communicator (Com), Medical Expert (ME), Manager (M), Collaborator (Coll), Professional (P), Scholar (S)	
Competences and sub-competences	CanMEDS domains of competences	LoS^a^
Diagnostic process and therapy planning (including adequate communication)	Com, ME, Coll, M	
- Capability to gather information about the patient including different information such as medical records, communication with patient and team and focused physical examination.		
- Capability to analyse given information.		
- Capability to make decisions with the patient about his/her further treatment and discharge from hospital in a time efficient way.		
- Capability to exchange information with the ward round team before and after the ward round, including documentation in patient’s record.		
- Capability to inform the patient about further treatment and discharge from hospital.		
Empathy (including adequate communication)	Com	
- Capability to recognize the necessity for empathy in physician-patient interaction.		
- Capability to be empathetic in physician-patient interaction, if necessary.		
Leadership skills (including adequate communication)	Com, Coll, M	
- Capability to involve the team in the ward round process.		
- Capability to assign tasks to team members.		
- Capability to lead the patient especially via communication.		
Management of difficult situations and faults (including adequate communication)	Coll, M	
- Capability to recognize, assess and react to interruptions of the ward round.		
- Capability to recognize, assess and react to ward round faults.		
- Capability to recognize and react to conflicts within the team in the course of the ward round.		
- Capability to recognize and react to conflicts with the patient in the course of the ward round.		
Organization competence (including adequate communication)	Com, Coll, M	
- Capability to ensure a structured ward round process including preparation, consultation with the patient and the team.		
- Capability to ensure a sufficient time management by adapting the duration of the ward round to patient’s needs as well as on the occurrences of the day, focusing on relevant aspects in physician-patient communication and avoiding interruptions.		
Professionalism (including adequate communication)	Com, Coll, P	
- Capability to ensure reliable behaviour towards the team and the patient.		
- Capability to ensure a respectful physician-patient relationship.		
- Capability to be aware of one’s own facial expression and gestures.		
- Capability to adapt one’s usage of language.		
Self-management	ME, Coll, S	
- Capability to assess own personal and professional limits, and to react if necessary.		
- Capability to assess own actions in a self-critical way.		
- Capability to remain calm and professional in difficult situations.		
Teaching and learning abilities (including adequate communication)	S	
- Capability to convey knowledge to students by involving them in the ward round process and discussing patient cases.		
- Capability to improve own knowledge through reflection of the ward round, in total or in specific cases.		
Assessment procedure	Conducting a ward round in a training environment with self- reflection and feedback.	

^a^LoS = Level of supervision: (1) Observation but no execution, even with direct supervision, (2) Execution with direct, proactive supervision, (3) Execution with reactive supervision, i.e., on request and quickly available, (4) Supervision at a distance and/or post hoc, (5) Supervision provided by the trainee to more junior colleagues [18]

Communication competence (including physician-patient and physician-team-communication) was spread across various competences. It was thus not regarded separately but included in collaborative clinical reasoning, leadership skills, organization skills, empathy, professionalism, self-management, management of difficult situations and faults, and teaching abilities.

The sub-competences of clinical skills and patient-management were also assigned to overlapping domains of competences. Error management was combined with management of difficult situations.

To ensure the typical link of EPAs to an established competence framework in a second step CanMEDS roles were assigned to each domain of competence to facilitate the identification in which role a trainee may need further training. The Level of supervision [18] were used as assessment category. Five levels are used to describe the trainees’ progress:

1. Observation but no execution, even with direct supervision.
2. Execution with direct, proactive supervision.
3. Execution with reactive supervision, i.e., on request and quickly available.
4. Supervision at a distance and/or post hoc.
5. Supervision provided by the trainee to more junior colleagues.

## Discussion

With this study we are able to give a detailed picture of what constitutes ward rounds in internal medicine. We found ward rounds to be a complex task and could contribute to illustrating ward rounds in a detailed manner.

### Ward round as a complex, multifarious task

The review of literature showed a broad spectrum of competences relevant for conducting an internal medicine ward round. Especially collaborative clinical reasoning, communication with patient and in the team, organization and teaching abilities were named in the majority of the included articles. However, there was a lack of a structured interprofessional approach to ward rounds.

### The detailed picture of ward round competences

#### Communication, organization and collaborative clinical reasoning as core-competences

In a large extent, the competences identified in this study correspond to aspects found in literature: the role of communication as key competence could be clarified once more. It was not only described in the direct physician-patient contact, but was proven to be strongly attached to all other ward round competences.

Again, in line with prior research, collaborative clinical reasoning and organization skills were regarded as particularly important [2, 3, 25, 26].

#### Need for a stronger implementation of teamwork and teaching skills on ward rounds

Further, it could be shown, that the demands of young German doctors are not necessarily consistent with prior studies. Opposed to recent studies e.g. [2, 10, 27] which pointed out the importance of team work, our sample regarded these aspects as less important: less than 30 % of the experts mentioned team work spontaneously as a ward round competence in the open interview part. Reasons could be constrained availability of nurses, organisational shortcomings and the limited number of students fulfilling clerkships in academic hospitals. However, in line with prior research, both the interviewed expert nurses and physicians e.g. [28] highlighted the importance of nurses as regular participants on ward rounds, as they have essential and specific knowledge about the patients and may increase patient care through close interprofessional collaboration. A good team interaction positively influences quality of care, patient satisfaction and acceptance of treatment as well as patients’ compliance [10, 29]. Participation of nurses in internal medicine ward rounds is claimed and desired to meet the standard [3] and is therefore strongly considered in the EPA.

Above this, our results indicate that education during ward rounds is perceived less important than expected. Even though the experts found teaching a relevant technical skills and communication as well as patient management important, they described patient care as the key goal of ward rounds. Similarly to nurses, students were reported to be irregular participants during ward rounds, which may explain our results. We however understand ward rounds as rich educational encounter not only for students but also for physicians [4]. Ward rounds serve a situated learning environment in which not only appropriate ward round manner can be imparted but also medical, social, organizational, ethical and economical contents. It should thus be part of physicians’ philosophy and we strongly encourage utilizing the potential ward rounds provide as encounters for teaching and learning.

### Proposal of an EPA “Conducting an internal medicine ward round”

Built upon the identified ward round competences and observable tasks we propose a description of an EPA “Conducting an internal medicine ward round”. It can be used to prepare medical students and physicians for the affordances of their first day of professional work on an internal medicine ward. Nine competences, 18 sub-competences and 62 belonging observable tasks could be described, which can be used as a guideline for young doctors.

### Limitations of the study

Due to methodology, the sample size was comparably small for nationally and internationally generalizable results. However, we observed a clear saturation effect and propose similarity for other German areas and other international countries with similar health systems. Our study shows that the EPA approach is feasible to picture complex situations like ward rounds going beyond clearly defined tasks with a consistent structure and clear responsibilities. Besides, the emerged EPA is founded on a sound empirical basis exceeding prior approaches that specified EPA through small group discussions. While reliability and internal validity of data were ensured by using coding scheme and the insurance of a satisfying intercoder reliability, external validity needs to be examined to allow for transferability of data to other hospitals in other regions.

## Conclusions

The EPA emerged from an interview study with expert physicians and nurses representing the broad field of internal medicine and hospital characteristics. Thus, it maps the perspectives from both professions regarding relevant competences and belonging tasks that a physician should possess and fulfil on his or her first day after graduation. Based on their vast amount of experience, the interviewed physicians and nurses provided a distinguished picture of aspects relevant for conducting ward rounds in internal medicine. Further research is needed to find similarities and differences between different specialisations in medicine (e.g. surgery) and to define core competencies that are needed in all specialities.

The suggested EPA provides a framework for integrating relevant skills in both undergraduate and graduate medical education and facilitates the assessment of students’ and physicians’ ward round competences through observable tasks and thus eases decisions about individuals’ readiness for conducting ward rounds in an objective way [21, 30]. Moreover, the EPA can be used as a tool for self-reflection and feedback and thus has a high and wide practical relevance.

For an easier handling in practice, a short version with all domains of competences (Table 3) as well an extended version with sub-competences and observable tasks (Additional file 2: Data S2) is provided.

## Abbreviations

EPA, entrustable professional activity; LoS, level of supervision

## Additional files

Additional file 1: Data S1. Interview schedule. (PDF 409 kb) Additional file 2: Data S2. Entrustable professional activity “Conducting an internal medicine ward round” – extended version. (PDF 280 kb)
