# Supplementary material for: Medical ward round competence in internal medicine – an interview study towards an interprofessional development of an Entrustable Professional Activity (EPA)
Source: BMC Med Educ. 2016 Jul 11;16:174. doi: 10.1186/s12909-016-0697-y (PMC4940908; doi:10.1186/s12909-016-0697-y)
Supplement: Additional file 1: Data S1. — Interview schedule. (PDF 409 kb) [file 12909_2016_697_MOESM1_ESM.pdf]

Interview schedule

Description of the study: We are conducting a study on internal medicine ward rounds and on relevant skills and abilities required for them. The aim of these interviews is to gain insights on how experienced physicians and nurses perceive a typical ward round. This information will then be used to establish a concept for a ward round training.

Handling of data and data privacy: The collected data will be kept in confidence and personal details will not be passed on. To anonymize data we use a code. For academic analysis and publication of results only the code will be used and data will be presented in a way that no inference to the person of reference is possible.

Progress of today's interview: First, I will ask you some questions concerning structural aspects of ward rounds. Then, I will ask you questions concerning the relevant skills and abilities needed when conducting a ward round.

1. Collection of demographic data

Demographic data	
Gender	male/female
Age	
Profession	physician; nurse
Wards previously worked at	
Professional position	resident, chief resident;<br>nurse manager, practical instructor in nursing
Duration of occupation in total/at this hospital (years)	
Number of ward rounds per week	

2. Interview

Process	
	**Could you please describe the procedure of a typical ward round beginning with the preparation of the ward round?**
In case this information was not given, specify with reference to these questions:	At what time does a ward round usually take place?
	How long does the ward round typically take? (in total/ per patient)<br>How do you allot time efficiently during ward round?
	Who is participating in ward rounds? (profession, number of persons)<br>Which role does interprofessionalism play for ward rounds?<br>What impact does the participation of nurses have for a ward round?
	In which phases can a ward round be subdivided? (e.g. before ward round – organization, in front of the door – consultation, in patients room – interaction with the patient, in front of the door – consultation)
	In which way does information exchange between the participants take place before, during and after the ward round?
	How does the ward round usually end? How is it generally brought to an end? (e.g. concluding discussion, immediate disbandment)<br>What is the aim of a ward round?<br>**Do the participants reflect the ward round afterwards? How?**

Residents tasks	
	**What function does the resident have in the course of the ward round?**<br>**Which specific tasks belong to that function?**<br>Could you name skills and abilities a resident therefore has to have?
Team- and self-related aspects	
**Decision making**	**Which decisions are usually made during a ward round?**<br>Who is responsible for making those decisions?
	**How are those decisions made? Is there a decision making process?**
	**Which sources of information are used for decision making?**<br>How is the team involved in decision making?<br>How is the patient included in decision making?
**Leadership/ Adaption**	**Which challenges is a resident facing when conducting ward rounds?**<br>Which challenges are posed by leading the team?<br>How can a resident include the nurse most effectively in the ward round?<br>Which other persons should be included in the ward round process? Is this actually happening in professional practice?<br>(<u>If the interviewee can't find an answer:</u> Think of organisational, personal, team-related or structural challenges.)<br>How do changing framework requirements lead to an adaption/change of the ward round situation?
	**How can those challenges be addressed?**<br><u>If answers are to concrete:</u> Which abilities does a resident need to address those challenges?
**Empathy**	**How important is a resident's empathy with a patient during the ward round?**<br>If this is classified as not important, why?
	How do you do this? (Please name some examples for situations in which empathy was important.)
	Can you remember an example, when you couldn't project yourself into a patients thought/ when it was difficult? How did you handle this?
	How can one learn to project oneself into patient's thoughts? Who have you learned it from? How do you teach it to your students?
	What effect does this empathy have on the patient?
**Dealing with faults/ learning from faults/ self-reflection**	**Which difficult situations can occur in the course of the ward round?**<br>Which disturbances and interruptions can occur during the ward round?<br>How can these disturbances be addressed?<br>How can difficult situations be handled?<br>What can the resident do to decrease the effect of these disturbances?
	**Which faults can happen in the course of the ward round? How do you address them?** (to the faults he/she is naming)<br>**When a previously made mistake becomes obvious in the course of the ward round, how would you react?** (e.g. you notice that an examination didn't take place)

	**How do you react when you yourself make a fault in the course of the ward round?** How would you notice it?<br>Would other team members (e.g. nurses) draw your attention to the fault? If yes, how do you react?<br>Are there any situations in which you ask others for help (colleagues, team members)? If yes, how would you do it?
Communication	
**Physician-patient (verbal)**	**How would you describe the physician-patient communication in the course of the ward round?**<br>How and to what extent you adapt your language use to the patient and/or the ward round team? (If interviewee can't answer the question: Could you give an example in which adaption of the language is necessary?)<br>Could you describe what the adaption looks like?<br>Which role does the clarification of technical terms have for the patient in the course of the ward round?<br>How and to what extent does communication impact the physician-patient relation?
	**How do you determine which information is relevant for the patient?**
	**How do you convey this information?**
	**How far can you assess the resilience of the patient?**
**Physician-patient (non-verbal)**	**How would you describe the non-verbal physician-patient communication in the course of the ward round?**
**Physician-team**	**How would you describe the communication with the team during the ward round?**<br>How would you describe the interactions with other participating physicians?<br>Is there any interaction between physician and nurse?<br>How does the communication between physician and nurse occur in the course of the ward round?
Ward round as a teaching environment	
**Teaching during ward rounds**	**How much is taught during a typical ward round?**<br>How much teaching should happen during a typical ward round?<br>**How could you conduct a ward round to impart knowledge to students?**
**Learning to conduct a ward round**	**How did you learn to conduct a ward round?**
Aspects of a successful ward round	
	**What characterises a successful ward round?**<br>- **for the physician**<br>- **for the nurse**<br>- **for the patient**<br>How can this be influenced by the physician?
**Closure**	We have talked about different aspects relevant for ward rounds. Is there anything you would like to add?
	Thank you very much for your time!
