# Supplementary material for: Medical ward round competence in internal medicine – an interview study towards an interprofessional development of an Entrustable Professional Activity (EPA)
Source: BMC Med Educ. 2016 Jul 11;16:174. doi: 10.1186/s12909-016-0697-y (PMC4940908; doi:10.1186/s12909-016-0697-y)
Supplement: Additional file 2: Data S2. — Entrustable professional activity “Conducting an internal medicine ward round” – extended version. (PDF 280 kb) [file 12909_2016_697_MOESM2_ESM.pdf]

EPA „Conducting an internal medicine ward round" – extended version

Discipline	Internal Medicine
Title	Conducting a ward round in internal medicine
Description	Conducting the daily ward round in an internal medicine department, starting with the preparation.
CanMEDS domains of competence	Communicator (Com), Medical Expert (ME), Manager (M), Collaborator (Coll), Professional (P), Scholar (S)

Domains of competences	Sub-competences and observable tasks	LoS* 1	2	3	4	5
Communication (Com)	Capability to communicate with the patient.					
	- Trainee collects information from the patient (e.g. poses open questions concerning patient's condition and needs, takes patient's history).					
	- Trainee involves the patient in the decision making process.					
	- Trainee informs the patient about further treatment/discharge from hospital.					
	- Trainee encourages the patient to ask questions.					
	- Trainee focuses on relevant topics and plans further conversation; if necessary separately from the ward round.					
	- Trainee assesses patient's need for information and adapts the amount and kind of imparted information.					
	- Trainee adapts his/her language to the patient (e.g. avoids technical terms).					
	- Trainee reacts adequately to faults by informing the patient about the fault.					
	- Trainee shows empathy when necessary (e.g. reflects patient's emotions, keeps professional distance, takes time for the patient, and shows concern).					
	- Trainee pays attention to patient's facial expression and gestures.					
	- Trainee is mindful of his/her own facial expression and gestures.					
	Capability to communicate with the ward round team.					
	- Trainee collects information from the war round team (e.g. asks nurse about patient-related information; exchanges views with attending physicians).					
	- Trainee exchanges views with the ward round team before and after the ward round/seeing each patient and asks their opinion.					
	- Trainee focuses on relevant topics.					

	- Trainee assigns tasks for example through communication with the nurses.					
	- Trainee reacts adequately to faults by accepting hints of possible faults, informing the chief resident and giving the reason of the fault.					
	- Trainee allows attendance of students and involves them in the ward round by asking questions.					
	- Trainee discusses patient cases with students to impart medical knowledge.					
Diagnostic analysis and therapy planning (Com, ME, Coll, M)	Capability to gather relevant information concerning the patient.					
	- Trainee reviews patient's record emphasizing on new test results.					
	- Trainee collects information from the patient (e.g. poses open questions concerning patient's condition and needs, takes patient's history).					
	- Trainee performs a focused physical examination.					
	- Trainee collects information from the ward round team (e.g. asks nurse about patient's condition; reviews nurses' documentation; exchanges views with attending physicians).					
	Capability to analyze gathered information.**					
	- Trainee combines gathered information.					
	- Trainee evaluates gathered information (e.g. information from the nurse, test results).					
	- Trainee pays attention to patient's facial expression and gestures.					
	Capability to plan further treatment with the patient.					
	- Trainee structures the decision making process (e.g. separates time-consuming or complex decision from the ward round).					
	- Trainee plans further treatment (e.g. evaluates therapy, gives orders for diagnostics and therapy, plans discharge from hospital)					
	- Trainee involves the patient in the decision making process.					
	Capability to exchange information.					
	- Trainee informs the patient about further treatment and discharge from hospital.					
	- Trainee exchanges views with the ward round team before and after the ward round/seeing each patient.					
	- Trainee records new information, e.g. to transfer information to absent team members.					
Empathy (Com)	Capability to act in an empathic way with the patient.					
	- Trainee recognizes the necessity for empathy in physician-patient interaction.**					

	- Trainee shows empathy when appropriate (e.g. reflects patient's emotions, keeps professional distance, takes time for the patient, shows concern).					
Leadership skills<br>(Com, Coll, M)	Capability to lead the ward round team.					
	- Trainee involves other team members in the ward round process (e.g. asks about their opinion).					
	- Trainee assigns tasks to members of the ward round team, e.g. through communication with the nurses.					
	Capability to lead the patient.					
	- Trainee communicates directively with the patient.					
	- Trainee encourages the patient to ask questions.					
	- Trainee assesses patient's need for information and adapts the amount and kind of imparted information to these needs.					
Management of difficult situation and faults<br>(Coll, M)	Capability to deal with interruptions.					
	- Trainee recognizes and assesses the relevance of interruptions.**					
	- Trainee reacts adequately to interruptions (e.g. pauses the ward round if necessary).					
	Capability to deal with faults.					
	- Trainee recognizes and assesses the relevance of faults.**					
	- Trainee reacts adequately to faults (e.g. accepts hints regarding potential faults, informs patient/chief resident, gives the reason for the fault and corrects the fault).					
	Capability to deal with conflicts within the ward round team.					
	- Trainee recognizes conflicts within the ward round team.**					
	- Trainee reacts adequately to conflicts within the ward round team.					
Organization competence<br>(Com, Coll, M)	Capability to determine the general framework of the ward round.					
	- Trainee conducts a well-structured ward round (e.g. prepares the ward round, follows a standardized procedure inside and outside of the patient's room)					
	- Trainee adapts the ward round process to the ward round team composition.					
	- Trainee utilizes adequate tools (e.g. trolley with medical records, notepads).					
	Capability to organize and use time efficiently.					
	- Trainee adapts duration of the consultation to the patient (e.g. longer when emotional or medical needs).					

	- Trainee adapts duration of the consultation/ the ward round to events of the day (e.g. shorter when several admissions).					
	- Trainees focuses on relevant topics and, if necessary, plans further conversation separately (incl. conversation with relatives or physicians of other disciplines).					
	- Trainee avoids interruptions (e.g. turns off the telephone, briefs the team to reduce interruptions to cases of emergency).					
Professionalism (Com, Coll, P)	Capability to act in professional way toward patient and team.					
	- Trainee ensures reliable behavior towards the ward round team and the patient.					
	- Trainee ensures a respectful physician-patient contact (e.g. protects patients privacy, meets patient eye to eye, focuses on the patient).					
	- Trainee is mindful towards his or her own facial expression and gestures.					
	- Trainee adapts his language use to the patient (e.g. avoids technical terms).					
Self-management (ME, Coll, S)	Capability to reflect on and control own actions.					
	- Trainee is aware of his/her professional limits (e.g. consults more experienced colleagues after the ward round in case of uncertainties or medical problems).					
	- Trainee is aware of his/her personal limits (e.g. asks for help in case of interpersonal problems).					
	- Trainee assesses his/her own actions (e.g. the interaction with the patient) in a self-critical way.					
	- Trainee remains calm and behaves professional in difficult situations.					
Teaching and learning abilities (S)	Capability to impart knowledge.					
	- Trainee allows attendance of students and involves them e.g. through asking questions.					
	- Trainee discusses patient cases with students to impart medical knowledge.					
	Willingness to improve own competences.					
	- Trainee reflects the overall ward round or specific cases after the ward round.					

* LoS = Level of supervision: (1) Observation but no execution, even with direct supervision, (2) Execution with direct, proactive supervision, (3) Execution with reactive supervision, ie, on request and quickly available, (4) Supervision at a distance and/or post hoc, (5) Supervision provided by the trainee to more junior colleagues (Ten Cate, 2013)

** Task is not directly observable, but listed here for completeness of content.
